# An interview study exploring healthcare professionals’ experiences of supporting health behaviors in migrant women after childbirth with special emphasis on mHealth

**DOI:** 10.1038/s41598-025-01147-3

**Published:** 2025-05-17

**Authors:** Maryam Shirvanifar, Ulrika Müssener, Alice Lindh, Josefin Wångdahl, Pontus Henriksson

**Affiliations:** 1 Department of Health, Medicine and Caring Sciences, Linköping University, Linköping, 581 83 Sweden; 2 Aging Research Center, Karolinska Institutet and Stockholm University, Stockholm, Sweden; 3 Department of Public Health and Caring Sciences, Uppsala University, Uppsala, Sweden

**Keywords:** Diet, Immigrant health, Physical activity, Practitioners, Qualitative study, Telemedicine, Disease prevention, Nutrition

## Abstract

Migrant health, including reproductive health, is a public health priority. The time after childbirth is considered an important period for health behavior change and mobile health (mHealth) interventions have shown promise in influencing health behaviors. Healthcare professionals have an important role in providing support for health behaviors and implementing mHealth tools in clinical care. This study therefore examined healthcare professionals’ experiences of health behavior promotion in migrant women and the potential for an mHealth intervention to improve health behaviors after childbirth. Twenty healthcare professionals in Sweden participated in individual semi-structured interviews. The informants had different professional backgrounds, ages, working experiences and all were females. Data was analyzed using thematic analysis and three main themes were generated. The first theme “Priorities and routines of health behavior promotion after childbirth” described a lack of priority for health behavior promotion after childbirth. The second theme “Social influences of health behaviors” described several factors that influenced possibilities for healthy behaviors after childbirth. In the third theme “mHealth in supporting health behaviors after childbirth”, informants stressed that a culturally appropriate smartphone app with reliable information had the potential to promote health behaviors after childbirth although further research is needed.

**Supplementary Information:**

The online version contains supplementary material available at 10.1038/s41598-025-01147-3.

## Introduction

Europe hosts many international migrants, with about 15% of the adult population born in a different country from where they currently reside (hereafter referred to as migrants)^1^. In Sweden, about 20% of the whole population and 25% of women of childbearing age are foreign-born^2^. Thus, migrant health is a crucial public health priority that affects millions of people globally including Europe^3–5^. Migrant women often face significant challenges in accessing healthcare services and achieving optimal health outcomes, including their reproductive health, compared to native-born women^5–9^. Nevertheless, it is important to note that migrants are a highly heterogeneous group. Factors such as socioeconomic and educational status, reason for migration, language barriers, the length of time spent in the new country and health behaviors may significantly influence health outcomes^4–6^. Hence, it is important to understand the factors that may contribute to poorer health and health behaviors among migrant women in high-income countries.

The period after childbirth is considered important for the future health of the mother^10,11^. For instance, weight retention and changes in diet quality after childbirth have been associated with a higher risk of obesity and cardiovascular disease risk later in life^12–15^. Thus, this period offers an opportunity for health behavior change and multiple studies show that various interventions could promote healthy body weight, diet and physical activity after pregnancy^11,16,17^. Studies have also shown the promise of mHealth interventions such as smartphone apps to promote healthy behaviors and weight gain during pregnancy^18^. However, there is a limited body of research about promoting health behaviors after childbirth through mHealth within healthcare^11,17^ especially in migrant women^19^.

Thus, there is a need for adapted and culturally appropriate mHealth tools to support healthy lifestyle behaviors in migrant women after childbirth and to examine the effectiveness of such tools. Healthcare professionals have an essential role in health behavior promotion, e.g., supporting healthy lifestyle behaviors such as a healthy diet, regular physical activity, weight management, smoking cessation, moderate alcohol consumption, adequate sleep, and stress management, in clinical care^20,21^ and in the implementation of mHealth tools into clinical practice^22^. However, to the best of our knowledge, no previous study has explored the perspectives from healthcare professionals regarding the potential of an mHealth intervention to promote health behaviors in migrant women after childbirth. This study therefore aimed to examine Swedish healthcare professionals’ experiences of health behavior promotion in migrant women and the possibilities and requirements for an mHealth intervention to improve health behaviors after childbirth.

## Materials and methods

### Study design

This qualitative study is part of the PRIMI (Promoting Reproductive health In MIgrant women) project which aims to develop and evaluate an mHealth intervention to promote healthy lifestyle behaviors in migrant women after childbirth. The qualitative interviews were intended to inform the design of this mHealth intervention and was guided by the Reflexive Thematic Analysis Reporting Guidelines (RTARG)^23^. Ethical approval for the study was granted from the Swedish Ethical Review Authority (reference number: 2022-06733-01) and all study procedures were conducted in accordance with the Declaration of Helsinki^24^.

### Study setting and recruitment

This study was conducted within the healthcare setting in Sweden including maternity clinics, healthcare centers and public health units. In Sweden, antenatal and postnatal care is offered through the public healthcare system and is free of charge. Pregnant women are typically followed by a midwife at a maternity clinic with approximately 8–9 routine visits during pregnancy. After delivery, care is transferred to child health services, where nurses and physicians monitor the health and development of the child and provide support to the mother and family. One or two routine postpartum visits with a midwife are also offered for mothers approximately 6–16 weeks after birth. Migrant and Swedish-born women are offered the same care within this system, although differences in healthcare utilization have been reported^25^. The study included four different healthcare regions in Sweden, each with a highly diverse population in terms of birth regions and educational backgrounds, encompassing both rural and urban areas. Purposive sampling was used to recruit healthcare professionals within these four healthcare regions to obtain a variety of occupational and professional experience and expertise. Inclusion criteria were healthcare professionals with experience in health promotion after childbirth in migrant women. These inclusion criteria were set to recruit informants with various professions, age, and experiences. Eligible informants were recruited by MS (Maryam Shirvanifar), AL (Alice Lindh) and KA (Kajsa André) and ML (Marie Leksell). Forty-three professionals were invited to participate. They received information about the study by e-mail prior to interviews, had the opportunity to raise questions, and were informed about the aim of the study and confidentiality. Those who were interested in participating registered their interest by email. Five declined to participate due to lack of time and 18 did not respond. None of the interviewers knew the informants before the study. The final sample therefore consisted of 20 healthcare professionals with a wide range of health professions (including midwife, dietician, medical doctor, and health communicator, Table 1). The informants worked in various healthcare settings, including maternity wards, obstetrics and gynecology clinics, midwifery clinics, child health centers, primary care centers, endocrinology clinics, public health centers, and within municipal services supporting newly arrived migrants. Their ages ranged from 28 to 68 years, and their work experience ranged from 2 to 38 years.

**Table 1:** Characteristics of the informants.

Informant	Profession	Clinical experience
A	Midwife	20 years
B	Healthcare administrator	4 years
C	Dietician	37 years
D	Midwife	26 years
E	Dietician	2 years
F	Healthcare strategy manager	8 years
G	Medical doctor	5 years
H	Psychologist	24 years
I	Dietician and operations manager	9 years
J	Specialist doctor	30 years
K	Midwife	15 years
L	Midwife	8 years
M	Midwife	6 years
N	Midwife	28 years
O	Manager for healthcare administration	38 years
P	Health communicator	15 years
Q	Psychotherapist	23 years
R	Health communicator	16 years
S	Psychologist	16 years
T	District nurse	12 years

### Data collection

An interview guide with semi-structured questions (Supplementary information) was developed by the authors, based on prior research and clinical experiences. The interview guide contained questions regarding the health professionals’ experiences of possibilities and challenges in promoting health in migrant women after childbirth as well as the requirements and potential of an mHealth intervention. The interview guide also included questions regarding health literacy. However, these findings will be presented in a separate manuscript in order to keep the main focus on health behavior promotion and mHealth development in the current study. At the end of the interview, informants were given the opportunity to provide additional information. The interview guide was tested in two pilot interviews and minor revisions regarding the formulation of some follow up questions were made after these interviews. These two pilot interviews were included in the analysis. All interviews were conducted in February and March 2023. Each informant was interviewed individually one time by MS, KA or AL. A total of nine interviews were conducted in person at a location selected by the informant (e.g. workplace or meeting room at a university) and eleven interviews were conducted via video call in the Zoom application. The interviews were audio recorded with a dictaphone and no video recordings were made. The duration of the interviews ranged from 23 min to 109 min (median: 57.5 min). Notes were taken during all interviews, and reflective notes were written immediately after each interview. Interviews were transcribed by a professional transcription company.

The research team comprised two female PhD students: MS, a registered dietician born outside Sweden with a master’s degree in international health, and ML, a medical specialist in orthopedics with a migrant background and previous experience in research. In addition, two female medical students (AL and KA) participated in the development of the interview guide and data collection process. The team also included a female associate professor (UM) with expertise in qualitative methodology and a female associate lecturer (JW) with wide experience in promoting migrant health and expertise in qualitative methodology. The principal investigator of the project was a male associate professor (PH) with previous experience in promoting health related to pregnancy and developing mHealth interventions including those targeting migrants.

### Data analysis

Reflexive thematic analysis as defined by Braun and Clarke^26,27^ was used to analyze the data as it offers a flexible yet theoretically grounded approach to identifying patterns of meaning across qualitative data. More explicitly, we applied a semantic approach of this analysis, as our primary goal was to explore healthcare professionals’ explicit experiences to inform the development of our mHealth intervention. Initially, agreement was verified between transcriptions and audio recordings. Potentially identifying details were changed or removed, and all informants were given pseudonyms to ensure confidentiality. The reflexive thematic analysis involved an iterative process between the following steps:

1. Familiarization with the data: MS and AL listened to all audio files and read the respective transcripts several times. Overall impressions, ideas and thoughts were noted.
2. Generating initial codes: MS and AL independently identified specific pieces of text or data that encapsulate a single idea or concept pertinent to the research aim which were used to derive initial codes. After individual coding, the codes were compared and discussed to deepen the analysis and to inform the subsequent generation of themes.
3. Generating themes: the codes were organized into patterns of shared meaning across the data, and from these, potential themes were generated by MS and UM.
4. Reviewing potential themes: themes were checked against the codes and transcripts, and refined through continuous discussions with MS, UM and PH.
5. Defining and naming themes: final themes were reached during several joint discussions with all authors.
6. Producing the report: themes were refined and ordered to present the results in a coherent and structured way. A narrative account was developed to communicate the results, supported by illustrative data extracts.

## Results

**Fig. 1:**
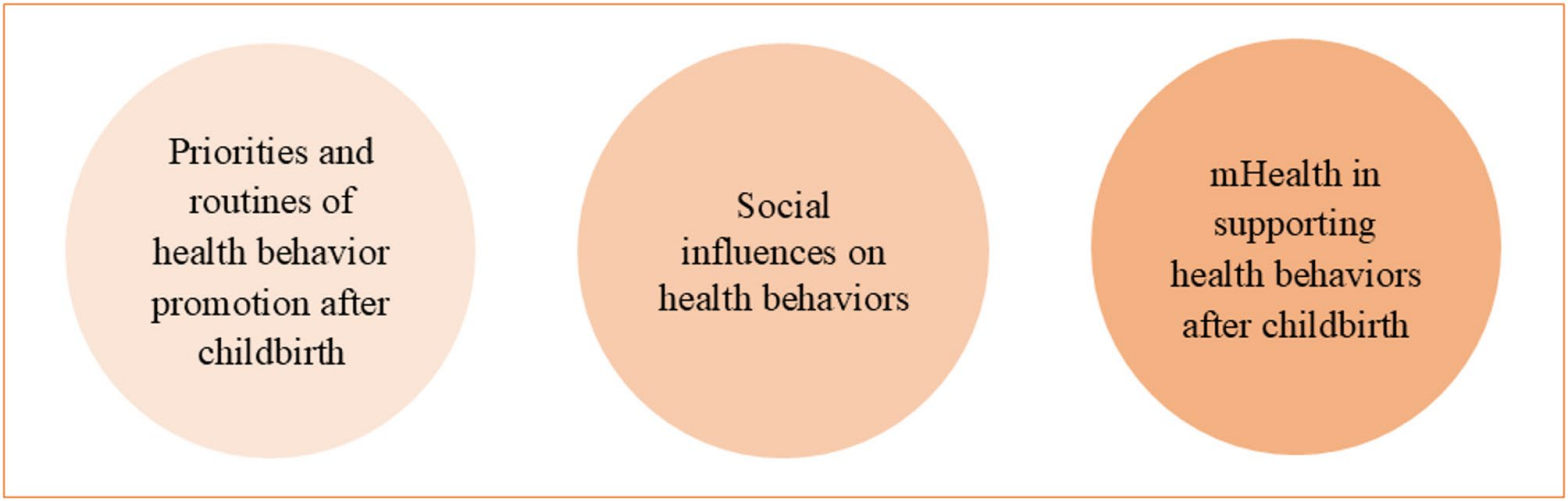
Themes from thematic analysis.

Three main themes were generated from the data: (1) Priorities and routines of health behavior promotion after childbirth, (2) Social influences on health behaviors, and (3) mHealth in supporting health behaviors after childbirth, as presented in Fig. 1.

### Priorities and routines of health behavior promotion after childbirth

Different aspects of priorities regarding health promotion targeting migrant women were revealed. One central aspect that was highlighted concerned priorities in resources and services. The informants claimed that care after childbirth risks falling between the cracks when it comes to health promotion. They explained that there is an uncertainty regarding who is responsible for health promotion since the care is shared between different professions within healthcare (e.g. midwives, physicians, nurses, dieticians, health coordinators) and sectors of healthcare.

*The postpartum period tends to be a kind of no-man’s land. There are many discussions at a strategic level. Who owns the question? It usually lands on which primary care you choose. There everyone looks at each other and wonders why we should work with healthy women.*

*Informant “F”*.

Informants also believed that the lack of priority for health behavior promotion after childbirth influenced the routines for such promotion. Thus, the informants expressed that the healthcare system in Sweden has a variety of routines for care after childbirth, but there is a lack of general routine monitoring of health behaviors of mothers after childbirth. Limited time in the meetings and shortages of staff were perceived to lead to the down-prioritizing of preventive care and healthy lifestyle advice. Informants also explained that available routine checkups after childbirth are neither adopted nor tailored specifically for the migrant women. The informants suggested that some migrant women may need extended resources and services like using interpreters, pictures and translated brochures in order to fully access and benefit from healthcare services. According to some informants, such customized support may be difficult to provide since it is time-consuming.

*I think it will be difficult for healthcare to provide* /…/ *It will probably take too much time to give individual advice to everyone.*

*Informant “E”*.

Furthermore, informants pointed out that shortcomings related to priorities and routines of health behavior promotion after childbirth might lead migrant women to seek information from other sources. These sources can include unverified internet sites, social media, or non-professionals around them, which are not always reliable and may lead to the uptake of inaccurate or potentially harmful advice.

*A lot of information may come from people in their social environment* /…/ *Unfortunately*,* that information is not always correct.*

*Informant “M”*.

Some suggestions for improving the priorities and routines of health behavior promotion after childbirth for migrant women were given. Communication between migrant women and healthcare workers could be improved through targeted cultural education for the healthcare staff. Improved communication between healthcare staff and patients was considered to enhance mutual understanding and trust and increase the chance of providing information in a personalized manner. Further, healthcare professionals with a migrant background could be important resources to assist or educate their colleague if they have the same cultural background and speak the same language as the patient.

*All of them [migrant health professionals] have been new in the country themselves and are language carriers. They have a large network of people that have migrated who have cultural competence in how to best convey information to a person with a different cultural background from Swedish. It’s an incredible resource.*

*Informant “B”*.

### Social influences of health behaviors

The informants believed that several factors linked to the social determinants of health affect health behaviors in migrant women. These factors were knowledge and language proficiency, economy, social network, and the prerequisites and transition of health behaviors in the migration process. All these factors were considered important and influenced the work toward promoting health behaviors in migrant women after childbirth.

Language barriers and lack of knowledge about the importance of health behaviors and where to seek help in the healthcare system were seen as factors that might reduce the opportunities for adopting healthy behaviors. Furthermore, diverse educational backgrounds of migrant women were thought to complicate meeting specific needs.

*It can be difficult to really give that knowledge so that they can adapt it individually to their own lives. Then it easily becomes one-fits-all advice. And that’s probably where it might become too generic and then it doesn’t fit and then you renounce it completely.*

*Informant “E”*.

Successful attempts for improving the knowledge in migrant women and minimize the language barriers are ongoing within healthcare. The informants explained that the family centers for instance, have groups in different languages, like “*mother-baby Swedish classes*” to provide comprehensive information for migrant women. These family centers also have health promotion sessions, talking about diet and physical activity in their own language, but they are not specific for the period after childbirth.

*At the family center*,* where they have had mother-baby Swedish classes for women who are at home with their babies. And there*,* we have had themes such as diet and physical activity. Such occasions should be held more often. And especially in their native tongue*,* so there will be a dialogue*,* a discussion around it.*

*Informant “P”*.

The economy was suggested to play a significant role in some migrant women’s lifestyle choices. Maintaining a healthy and balanced diet with nutritious foods like fresh produce, whole grains, and low-sugar options is costly. Despite the large heterogeneity in the migrant population, many migrant families live on low incomes, and larger families face even greater challenges. This also applies to the possibilities of engaging in physical activities, as a gym membership, or suitable sportswear are also costly.

*There I also believe that the economy serves as an obstacle.* /…/ *Yes*,* it does* /…/ *It is an obstacle if there are many people in the family. Even if you know how to do it.*

*Informant “I”*.

Belonging to a group or a social network was another aspect of importance related to health promotion targeting migrant women. The informants pointed out the “*sistership*” between migrant women and believed using these networks with a focus on promoting a healthy lifestyle would be motivating to make changes. Observing fellow members engaging in exercise for example, could serve as a motivational catalyst, generating a positive domino effect according to our informants.

*You take after one another. If you see that there are others around you doing it, then it becomes that you can do it and must also do it.*

*Informant “P”*.

Further, the influence of migrant women’s backgrounds on health behaviors was raised. This includes the transitioning from habits in their birth countries to those in their new home country, which may not always be the same.

*I come from another country and the cultural thing is that you should preferably be at home 40 days after you have had a baby* /…/ *Then I met someone I knew*,* that thought that it was very strange. “What are you doing outside? You should be at home. You should not go out at all*,* you should be at home.*

*Informant “P”*.

### mHealth in supporting health behaviors after childbirth

The informants believed that a smartphone app could possibly support the provision of health information in an accessible language and help users set goals for their progress in promoting lifestyle habits. They thought that an app could be beneficial for migrant women to easily access health advice. Furthermore, informants suggested such an app should contain accurate and accessible educational modules about the human body, nutrition, physical activity, and the impact of a healthy lifestyle on health, with well-translated explanations and helpful visuals for easy comprehension. The need for reliable information was stressed.

*I see benefits in referring to something that has reliable information.*

*Informant “E”*.

Further, the importance of a user-friendly and easy to use smartphone app was highlighted. To involve and engage women with limited literacy skills, informants suggested incorporating videos, images and an audio feature for listening to information instead of reading it. The lack of time due to family commitments was seen as a common reason migrant women were unable to attend physical appointments. The possibility of getting information through a smartphone app was seen as a way to improve accessibility to health information and promotion.

*The women can watch for themselves at home, on their phones, when they have time and are looking for knowledge. All that we don’t have time for here (maternal health center), they can get themselves.*

*Informant “N”*.

Features like the active input of personal data like body weight or blood sugar were assumed to enhance the relationship-building with healthcare professionals for migrant women and to engage them in monitoring their own health. Informants explained that some migrant women would probably appreciate receiving advice anonymously, in order to seek help or find reliable information without telling anyone or without visiting healthcare. Further, motivating notifications and an awarding system like getting stars or medals, with achievable challenges (e.g. short walks with health advice about its advantages) and daily advice were mentioned and seen as beneficial for using the app regularly.

*So, I think you could probably click there for notifications. Now, you should go for a 30-minute walk.*

*Informant “K”*.

Finally, informants described that using relatable images of people from similar backgrounds and recommendations in the form of familiar healthy foods that migrant women can relate to, were considered to make the smartphone app more inclusive and culturally appropriate which in turn may enhance the engagement levels.

*Adapted to their cultural background*,* not Swedish information translated into Arabic; someone who knows how their regular diet looks*,* something confirmed by people who know what* they *are talking about regarding lifestyle.*

*Informant “E”*.

## Discussion

This qualitative study examined healthcare professionals’ experiences of health behavior promotion in migrant women and the possibilities and requirements for an mHealth intervention to improve health behaviors after childbirth. We found that the informants experienced a lack of priorities and routines for promoting health behaviors after childbirth which was a major challenge. Furthermore, although informants highlighted the heterogeneity in the migrant population, they also experienced that several factors such as knowledge and language proficiency, economy and social networks influenced the possibilities of having healthy behaviors after childbirth. Finally, they believed that a culturally appropriate smartphone app with reliable information has the potential to promote health behaviors after childbirth.

A significant finding in our study was that the informants reported an absence of prioritization and established routines for monitoring and promoting health behaviors after childbirth for mothers, including migrant women. This is critical as the period after childbirth represents a crucial phase for promoting health behaviors, particularly in terms of diet and physical activity^11,16,17^. According to the informants, women require robust familial and social support to manage their weight, adopt a healthy diet, and engage in physical activities during this time. Addressing these challenges and actively promoting health behaviors and healthy body weight among women after childbirth may reduce the risk of negative health outcomes such as obesity and cardiovascular disease later in life^12–15^.

Cultural competence which is a term commonly used in the healthcare context^28^, has been defined as “a set of congruent behaviors, attitudes and policies that enables healthcare workers to work and communicate effectively and appropriately in cross-cultural situations”^28,29^ The informants believed that the cultural competence of the healthcare system could be enhanced by taking advantage of the diverse backgrounds of the healthcare professionals within the system. Further, they believed that such awareness was important to tailor health behavior promotion to better meet the specific needs of migrant women. Previous studies examining reproductive health in migrant women have also suggested the cultural competence training is important for understanding diverse perspectives and cultural norms to facilitate effective and empathic communication^30,31^.

The informants pointed out several other factors they believed influenced health behaviors, although they also highlighted the heterogeneity within the population of migrant women. For instance, they mentioned that language barriers, lack of knowledge about the importance of health behaviors, and uncertainty about where to seek help in the healthcare system seem to influence migrant women’s ability to adopt a healthy lifestyle. This is in line with several previous studies highlighting linguistic challenges as a barrier to delivering adequate and equitable healthcare to migrant women^30,32–34^. In a review focusing on migrant women in Europe and their need for pregnancy-related care, a common theme across most studies was the issue of insufficient information and communication which could influence access to maternity services^35^. Previous studies suggest that lack of knowledge about the importance of health behaviors^32,36^ and where to seek help in the healthcare system^31^ might reduce health-seeking behavior, which in turn may limit the opportunities to receive health behavior promotion within clinical care. Economy was another factor mentioned by the informants as significant in adapting health behaviors like a healthy diet and physical activity which is relevant since income/economy is well-recognized as a social determinant of health^37^.

Informants believed a smartphone app had potential to promote a healthy diet and physical activity after childbirth, either independently or in combination with physical meetings. They also noted that the app could provide accessible, reliable, evidence-based information in multiple languages. To the best of our knowledge, no previous qualitative study has explored the potential of an mHealth intervention to promote health behaviors in migrant women after childbirth. However, our results may be compared to such studies during pregnancy. One study reported that additional information delivery methods (such as audio or video-files) may be useful for the creation of a smartphone app accessible to a diverse range of women^31^. A review of pregnancy apps, with emphasis on culturally and linguistically diverse women, highlighted the need for reliable information from credible sources as well as the importance of representing women with diverse backgrounds to ensure inclusivity^38^. These aspects will be relevant to consider when developing a smartphone app to promote health behavior in migrant women after childbirth. Future development could incorporate participatory design involving the target group (i.e. migrant women after childbirth)^39^ to inform the design of such a smartphone app. It is also important to consider person-centered care, cultural sensitivity, and health literacy responsiveness in the app’s development. In addition, it is crucial to assess the feasibility of implementation, including the settings and clinical workflows in which healthcare professionals would deliver the intervention. Moreover, it is important to consider the logistical challenges and opportunities for implementing mHealth tools into the existing healthcare system, such as staff training, digital infrastructure, and time constraints in clinical practice. These challenges have also been identified in a systematic review which reported that technical (e.g., compatibility with workflow and other systems, concerns about regulation and security), individual (e.g., resistance to change, limited knowledge regarding the technology), and healthcare system barriers (e.g., lack of policies and standards, economic and legal factors) may hinder the adoption of mHealth technology^40^. Thus, it is important that the implementation of mHealth tools into maternal health services is further explored as part of future research and pilot testing. Finally, although the informants observed potential for smartphone app interventions to promote health behaviors in migrant women after childbirth, future studies are warranted to evaluate the effectiveness of such interventions. Additionally, future studies should explore the perceptions of migrant women to ensure the app meets their needs and preferences.

### Methodological considerations

A qualitative design^41^ was considered advantageous in gaining comprehensive insights into the perspectives of healthcare professionals about promoting a healthy lifestyle among migrant women. Several procedures were taken to fulfil the quality criteria for qualitative research; dependability, credibility and transferability to verify trustworthiness. To attain dependability, the research process was clearly described and reported^42^. Furthermore, it was guided by the Reflexive Thematic Analysis Reporting Guidelines (RTARG)^23^, which emphasize transparency and reflexivity throughout the research process. We used a semi-structured interview guide for systematic data collection^43^ and followed Braun and Clarke’s stages for reflexive thematic analysis^26,27^ facilitating a structured and systematic process. The number of participants was guided by information power, which refers to the principle that the more relevant information a sample holds for addressing the study aim, the fewer participants are needed^44,45^. More specifically, fewer participants are required when the study has a relatively narrow aim, high sample specificity, is guided by previous theory or knowledge, and includes strong quality of dialogue, as in our study. Accordingly, data collection was finalized when we believed that our analysis of the transcripts had reached satisfactory depth and complexity to answer the research questions. The credibility of the analysis was supported through reflexive engagement, where two researchers independently coded the data and engaged in collaborative discussions with the research team to critically explore and refine the themes. In addition, to ensure transparency, excerpts from the transcribed texts are included as representative quotations^43^. The data were collected from informants from four different healthcare regions in Sweden who had experience working with migrant women. Including diverse professions, ages and clinical experiences enriched the data with different perspectives. Furthermore, some informants were born outside Sweden, offering valuable insights relevant to the aim of the study. While the transferability of results to other regions may vary, depending on how care is organized, we consider the findings applicable to developing mHealth interventions to improve health behaviors after childbirth in similar settings.

Noteworthy, our study had some important limitations to consider. Informants were purposefully recruited and were only female. The uneven distribution of men and women might limit the diversity of perspectives and experiences in our data. However, this is reflective of the maternal care profession in Sweden, for instance only 0.3% of midwives in Sweden were male in 2019^46^. Finally, this study solely explored healthcare professionals’ experiences of health behavior promotion in migrant women after childbirth. Although the perspectives of healthcare professionals are essential to improve clinical care, future studies within this project (the PRIMI project) will examine the corresponding perspectives of migrant women to ensure that all perspectives are comprehensively examined.

## Conclusion

There is a lack of priorities and routines for health behavior promotion after childbirth in migrant women and cultural competence may improve such health promotion. Furthermore, several factors such as knowledge and language proficiency, economy and social networks may influence the possibilities of having healthy behaviors after childbirth although there is considerable heterogeneity within the migrant population. Finally, informants believed that a culturally appropriate smartphone app with reliable information might have the potential to promote health behaviors after childbirth. However, further studies are warranted to examine the effectiveness of such an mHealth intervention to promote health behaviors in migrant women after childbirth.

## Electronic supplementary material

Below is the link to the electronic supplementary material.

Supplementary Material 1

## Data Availability

To safeguard the identities of informants, complete data (transcribed interviews and audio files) will not be made available to the public. However, transcribed interviews may be available from the corresponding author on reasonable request.
