# Supplementary material for: An interview study exploring healthcare professionals’ experiences of supporting health behaviors in migrant women after childbirth with special emphasis on mHealth
Source: Sci Rep. 2025 May 17;15:17208. doi: 10.1038/s41598-025-01147-3 (PMC12085631; doi:10.1038/s41598-025-01147-3)
Supplement: Supplementary file 1 — Supplementary Material 1 [file 41598_2025_1147_MOESM1_ESM.pdf]

SUPPLEMENTARY MATERIAL

Supporting health behaviors in migrant women after childbirth with special emphasis on mHealth:

An interview study with health care professionals

Maryam Shirvanifar[1], Ulrika Müssener[1], Alice Lindh[1], Josefin Wångdahl[2,3], Pontus Henriksson[1]

[1] Department of Health, Medicine and Caring Sciences, Linköping University, Linköping, Sweden

[2] Aging Research Center, Karolinska Institutet and Stockholm University, Stockholm, Sweden

[3] Department of Public Health and Caring Sciences, Uppsala University, Uppsala, Sweden.

Corresponding authors: Pontus Henriksson and Maryam Shirvanifar, Department of Health, Medicine and Caring Sciences, Linköping University, 581 83 Linköping, Sweden.

E-mail: pontus.henriksson@liu.se and maryam.shirvanifar@liu.se.

Telephone number: +4613-28 10 00.

Interview Guide

I. Health promoting work in migrant women after childbirth

- How would you describe your role in working with migrant women regarding lifestyle and health?
- What responsibility do you think the healthcare system has in promoting a healthy lifestyle after childbirth?
- What opportunities does your professional role offer to promote healthy lifestyle habits after childbirth?
- What opportunities and challenges do you face in your professional role regarding the promotion of healthy lifestyle habits in migrant women after childbirth?
- In what ways is information and support offered to women to help them change their lifestyle after childbirth?
- How do you perceive the opportunities and challenges migrant women face in eating healthily and being physically active after childbirth?

II. Health literacy in migrant women after childbirth

- What role do you see the healthcare system playing in improving migrant women's ability to understand and use health-related information?
- What role do you think your professional role has in improving migrant women's ability to understand and use health-related information?
- Where do you perceive that women obtain health information?
- What opportunities and challenges do you perceive migrant women face in understanding health information?
- What challenges do you perceive that migrant women face in using the information you provide to them?
- What do you think could improve migrant women's ability to obtain and understand health information?

III. mHealth app in migrant women after childbirth

- What information and features do you think are needed in an app to promote a healthy lifestyle for migrant women after childbirth?
- What opportunities and challenges do you see with such an app?
- What is important to consider for the app to be functional linguistically and culturally for the women it is aimed at?

IV. Other

- Is there anything else you wonder about or would like to add?
